# In Vivo Target Engagement Assessment of Nintedanib in a Double-Hit Bleomycin Lung Fibrosis Rat Model

**DOI:** 10.3390/ijms27010064

**Published:** 2025-12-20

**Authors:** Vanessa Pitozzi, Paola Lorenza Caruso, Silvia Pontis, Barbara Pioselli, Francesca Ruscitti, Maria Gloria Pittelli, Costanza A. M. Lagrasta, Federico Quaini, Antonella Maria Nogara, Giancarlo Aquino, Roberta Volta, Maria Laura Faietti, Martina Bonatti, Paolo Spagnolo, Marcello Trevisani

**Affiliations:** 1 Chiesi Farmaceutici S.p.A., Global Research and Preclinical Development Area, Largo Belloli, 11/A, 43122 Parma, Italys.pontis@chiesi.com (S.P.); b.pioselli@chiesi.com (B.P.); f.ruscitti@chiesi.com (F.R.); m.pittelli@chiesi.com (M.G.P.); g.aquino@chiesi.com (G.A.); r.volta@chiesi.com (R.V.); ml.faietti@chiesi.com (M.L.F.); m.trevisani@chiesi.com (M.T.); 2 Department of Medicine and Surgery, University of Parma, 43126 Parma, Italy; costanzaannamaria.lagrasta@unipr.it (C.A.M.L.); federico.quaini@unipr.it (F.Q.); antonellamaria.nogara@unipr.it (A.M.N.); 3 Respiratory Medicine Unit, Department of Medicine & Center for Molecular Medicine, Karolinska Institute, 17176 Stockholm, Sweden; martina.bonatti@ki.se; 4 Respiratory Disease Unit, Department of Cardiac Thoracic, Vascular Sciences and Public Health, University of Padova, 35128 Padova, Italy; paolo.spagnolo@unipd.it

**Keywords:** Nintedanib, idiopathic pulmonary fibrosis, VEGF, bleomycin

## Abstract

Nintedanib is an anti-fibrotic medication endowed with a multi-kinase inhibitor profile and approved for the treatment of Idiopathic Pulmonary Fibrosis (IPF). Nintedanib is believed to inhibit mainly Vascular Endothelial Growth Factor (VEGF), Platelet-Derived Growth Factor (PDGF), and Fibroblast Growth Factor (FGF) receptor kinases. The main objective was to identify potential tissue and/or circulating biomarkers to demonstrate Nintedanib’s target engagement and support its in vivo pharmacodynamic activity, consistent with its proposed mechanism(s) of action. In four independent experiments of bleomycin (BLM)-induced lung fibrosis model in rats, animals received Nintedanib (oral, 100 mg/kg/day) from day 7 post-BLM for 3 weeks. As expected, Nintedanib significantly reduced lung weight, the levels of lung fibrotic markers, and fibrotic areas. Moreover, Nintedanib-treated animals expressed lower levels of FGF2 in lung homogenates and higher plasma and lung levels of VEGF (≥3-fold, *p* < 0.05) compared to control animals. Lung proteomic analysis revealed the inhibition of receptor tyrosine kinases signaling in Nintedanib-treated animals. Circulating and lung levels of Nintedanib confirmed an optimal tissue distribution in the rat, consistent with the data reported for humans. Although VEGF ligand levels are elevated in the lungs of Nintedanib-treated animals, the VEGF signaling pathway remained functionally downregulated, strongly suggesting compensatory VEGF feedback delivery to its receptor blockade by Nintedanib. In summary, based on the present experimental findings in rats and supporting clinical preliminary evidence, increased VEGF levels can be reasonably considered an indicator of target engagement for Nintedanib and potentially for other VEGF modulators.

## 1. Introduction

Idiopathic Pulmonary Fibrosis (IPF) is a deadly interstitial lung disease of unknown etiology characterized by poor quality of life, substantial morbidity, and significant healthcare resource utilization [1]. IPF affects the alveoli primarily before spreading to the surrounding lung tissue, which becomes thick and stiff, leading to irreversible architectural distortion [2]. Although the precise etiopathogenetic mechanisms underlying IPF remain elusive, current hypotheses suggest that the disease arises from repetitive microinjuries to a genetically susceptible alveolar epithelium. These injuries trigger an aberrant wound-healing response, marked by dysregulated fibroblast activation, differentiation to myofibroblasts, and excessive extracellular matrix deposition, particularly collagen [3].

Although two antifibrotic drugs, Pirfenidone (http://www.esbriet.com) and Nintedanib (http://www.ofev.com), have been approved for the treatment of IPF based on their ability to slow functional decline and disease progression [4], a definitive cure is not available, and clinical conditions in most patients continue to decline despite treatment. Consequently, there is a pressing need for the development of more efficacious and better-tolerated drugs [5,6]. Pirfenidone is a pleiotropic pyridine compound with an incompletely understood mechanism of action. However, it may act by modulating fibrogenic growth factors, thereby attenuating fibroblast proliferation, myofibroblast differentiation, collagen synthesis, oxidative stress, and deposition of extracellular matrix (ECM) [7,8]. Proposed molecular targets of Pirfenidone include p38 MAPK (Mitogen-activated protein kinase), TNF (Tumor necrosis factor), TRPA1 (Transient receptor potential ankyrin 1), the Wnt pathway, and TGF-β1 (Transforming growth factor) [9,10,11]. Nintedanib is an orally available, small-molecule multi-kinase inhibitor that targets multiple receptor tyrosine kinases (RTKs) involved in fibrosis, inflammation, and angiogenesis. Specifically, it acts as VEGF, PDGF, and FGF receptors modulator [12]. By inhibiting the activity of the above RTKs, Nintedanib attenuates the impact of various growth factors and cytokines involved in fibrosis and inflammation. In rodents, Nintedanib reduces pulmonary and extra-pulmonary fibrosis, suggesting that it may be able to exert its multi-target inhibitor effect also in animals [13,14,15]. Despite its proven efficacy, no analyses have been conducted in preclinical models to identify in vivo pharmacodynamic biomarkers and pharmacokinetics in relation to target engagement. As such, objective molecular preclinical readouts confirming the interaction between Nintedanib and its intended targets remain to be established.

To address this knowledge gap, we aimed to identify tissue and circulating biomarkers supporting Nintedanib’s target engagement and anti-fibrotic effect in a double-hit bleomycin rat model of lung fibrosis, while leveraging proteomic analysis of key markers involved in VEGF, PDGF, and FGF2 signaling pathways known to be modulated by Nintedanib [16]. Secondly, we quantified Nintedanib concentrations in both plasma and lung tissue to determine the pharmacologically active levels associated with its in vivo target engagement and anti-fibrotic efficacy.

## 2. Results

### 2.1. Kinase Cell-Free Assay

Three concentrations (100, 1000, and 5000 nM) of Nintedanib were counter-screened in a competitive assay against the above-mentioned pre-selected panel of kinase receptors. At 100 nM, Nintedanib exhibited full interaction with all PDGF and VEGF receptor kinases but with a weaker signal vs. FGFRs (% of the control response between 49 and 98). At higher concentrations (1000 and 5000 nM), Nintedanib interaction was also clearly captured against the four FGF kinase receptors (% of the control response between 0.1 and 17 for the 5000 nM) (see Table 1).

### 2.2. Therapeutic Effects of Nintedanib on BLM-Induced Pulmonary Fibrosis

**Figure 1:**
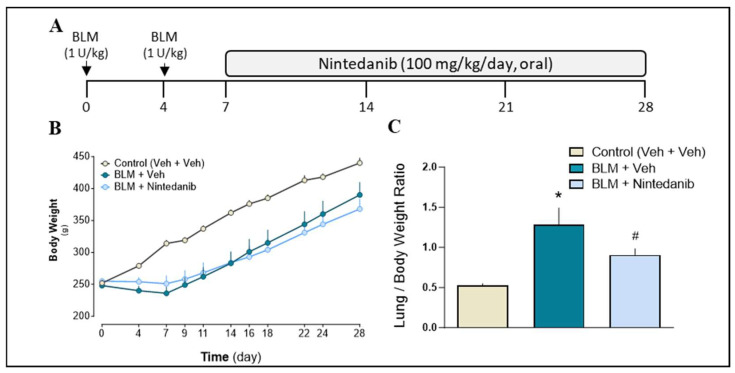
Experimental protocol. (**A**) Rats received a double intratracheal administration of BLM (1 U/kg) on day 0 and day 4. Nintedanib (100 mg/kg, oral) was administered once daily from day 7 to day 28. (**B**) Changes in body weight during the entire experimental protocol. (**C**) Lung/body weight ratio in control and experimental animals. Statistical analysis was performed with one-way ANOVA followed by Dunnett’s test. A *p*-value < 0.05 was considered statistically significant. * *p* < 0.05 when compared to the control group (cream column). # *p* < 0.05 when compared to BLM + Vehicle group (teal blue column).

To evaluate the effects of Nintedanib on BLM-induced pulmonary fibrosis, Sprague–Dawley (SD) rats were intratracheally injected with a double administration (double-hit) of BLM (1 U/kg). According to a therapeutic approach, Nintedanib (100 mg/kg/day in methylcellulose 0.5%) or its vehicle was orally administered for three consecutive weeks starting from day 7. Animals were sacrificed on day 28 for further analysis (Figure 1A).

#### 2.2.1. Animals General Health Condition and Body Weight

BLM administration led to a slowing in body weight gain, when compared to control animals, reaching a peak at day 7 (Figure 1B). Nintedanib was well tolerated. The body weight gain curves of BLM-treated animals with or without Nintedanib were virtually identical. The lung/body weight ratio of BLM-treated rats significantly increased compared to controls (likely due to increased levels of inflammation, fluid accumulation, matrix deposition, and fibrosis). Nintedanib significantly reduced the lung/body weight ratio by ≈50% (Figure 1C). No mortality was observed among the four independent experiments.

#### 2.2.2. Effect of Nintedanib on Markers of Fibrosis

**Figure 2:**
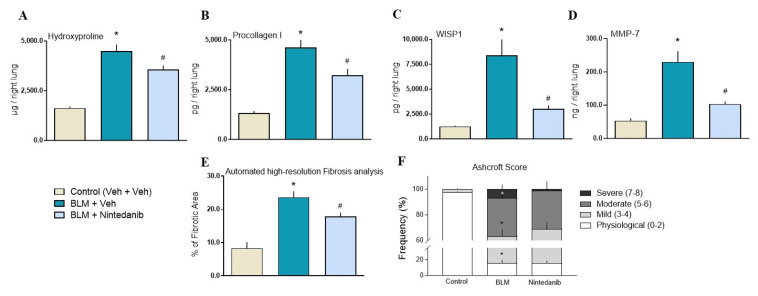
Evaluation of the anti-fibrotic activity of Nintedanib. Nintedanib (light blue columns) reduced multiple fibrotic markers measured by colorimetric or Enzyme-Linked Immunosorbent Assay kits. (**A**) Hydroxyproline; (**B**) Procollagen I; (**C**) WISP1; (**D**) MMP-7, in rat lung homogenate supernatants compared to their respective BLM groups (teal blue columns). Panel E and F show the inhibitory effect of Nintedanib on lung fibrosis analysis (**E**) and on Ahsctoft scores (**F**), measured on Masson’s trichrome-stained slides, relative to animals treated with BLM only. Statistical analysis was performed with one-way ANOVA followed by Dunnett’s test. A *p*-value < 0.05 was considered statistically significant. * *p* < 0.05 when compared to the control group. # *p* < 0.05 when compared to BLM + Vehicle group.

**Figure 3:**
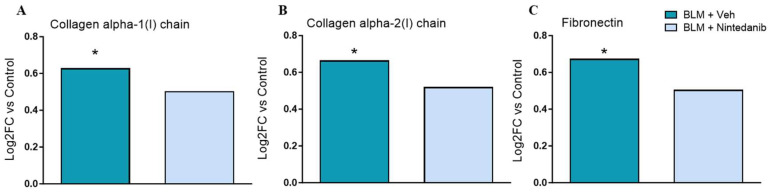
Proteomics evaluation of fibrotic markers. BLM up-regulated both Collagen type I alpha-1 chain (**A**) and alpha-2 chain (**B**), as well as Fibronectin (**C**), when compared to the control. Nintedanib was able to reduce the expression of the three fibrotic markers. Statistical analysis was performed with a *t*-test. * adj.*p* < 0.05 and log2FC > 0.58 when compared to Control group.

BLM induced a significant increase in all assessed markers of lung fibrosis: hydroxyproline (HYP), proCollagenI, WNT1-inducible-signaling pathway protein 1 (WISP-1), and matrix metalloproteinase-7 (MMP7). The levels of all markers were significantly reduced in Nintedanib-treated animals. Specifically, Nintedanib exhibited a greater inhibitory effect against WISP1 and MMP-7 (75% and 72% of reduction, respectively) (Figure 2A–D). Mass spectrometry (MS)-based proteomics of BLM-treated rat lung documented a significant rise in Collagen type 1 and Fibronectin, which were significantly reduced by Nintedanib (Figure 3A–C).

#### 2.2.3. Histopathological Analysis

At day 28, following double-hit BLM, histological examination of the lungs revealed partial loss of alveolar architecture and the presence of fibrotic lesions with ECM deposition. The quantification of fibrotic tissue by automated analysis confirmed a significant increase in lung parenchyma occupied by fibrosis in BLM-treated rats compared to controls. Lung fibrosis was significantly reduced by Nintedanib (37% reduction, *p* < 0.05, Figure 2E). Moreover, Nintedanib significantly reduced the frequency of the most severe Ashcroft scores of fibrosis, whereas its effect on mild fibrosis scores only trended towards significance (Figure 2F).

#### 2.2.4. Effect of Nintedanib on Presumptive Targeted Growth Factors in Lung Homogenate Supernatant and Plasma

**Figure 4:**
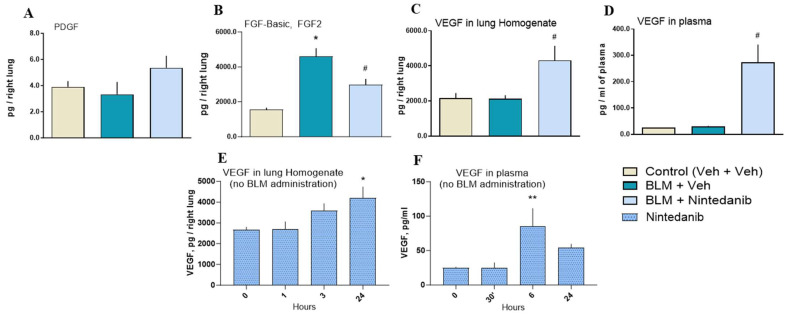
Assessment of Nintedanib target engagement. Nintedanib (light blue columns) did not modify lung levels of PDGF (**A**), which was unaffected by BLM, while significantly attenuated FGF2 lung levels (**B**) were enhanced by BLM. In contrast, lung (**C**) and plasma (**D**) VEGF levels were significantly increased by Nintedanib treatment relative to both untreated (control) and BLM treated groups. Panel (**E**,**F**) illustrates the rise in VEGF levels in lung and plasma, respectively, observed at different time points following Nintedanib treatment in BLM-free animals. Statistical analysis was performed with the one-way ANOVA followed by Dunnett’s test. A *p*-value < 0.05 was considered statistically significant. * *p* < 0.05, ** *p* < 0.01 when compared to the control group (cream columns, Panels **A**–**D**) or time points 0 (Panels **E**,**F**). # *p* < 0.05 when compared to BLM + Vehicle group (teal blue columns, Panels **A**–**D**).

Neither BLM nor Nintedanib treatments altered the lung levels of PDGF ligands (Figure 4A), while BLM increased FGF2 lung levels that were significantly reduced by Nintedanib when compared to control animals (Figure 4B). In contrast, Nintedanib significantly increased the lung and plasma levels of VEGF ligands compared to BLM-treated animals not receiving Nintedanib (Figure 4C,D). To dissect the implication of specific targeted engagement, we added a group of BLM-free mice treated with Nintedanib. Even a single oral administration of Nintedanib (100 mg/kg) in BLM-free animals was able to increase lung and plasma levels of VEGF, although with different kinetic profiles (Figure 4E,F).

### 2.3. Lung Tissue Phosphoproteomic Assessment in Nintedanib Naïve Animals

**Figure 5:**
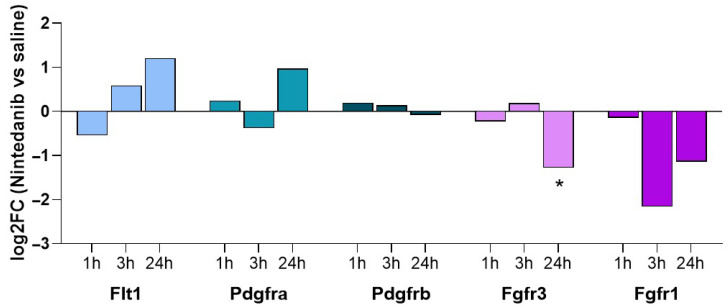
Proteomics evaluation of growth factor receptors. Fgfrs show a declining trend as a result of Nintedanib administration, while a time-dependent increased expression was observed for Pdgfra and Flt1. Statistical analysis was performed with a *t*-test. * adj.*p* < 0.05 and FC > 1.5 when compared to naive group.

A quantitative label-free phosphoproteomic analysis was performed on lung samples from BLM-free rats, untreated or treated with Nintedanib for 1 h, 3 h, or 24 h. All conditions were assayed in biological triplicate. The analysis identified 6645 proteins as master proteins with high confidence identification. A differential expression analysis was performed on quantified proteins, comparing Nintedanib-treated rats with untreated rats at each time point. The levels of VEGF, FGF, and PDGF were undetectable; however, the respective receptors were quantified. Flt1, one of the VEGF receptors, showed a trend of increasing concentration, although it did not appear to be significantly regulated by Nintedanib. Similarly, PDGFRA and PDGFRB did not show any significant regulation by Nintedanib. FGFR1 and FGFR3 proteins displayed a trend of a time-dependent decline, which reached statistical significance at 24 h, at least for Fgfr3 (Figure 5).

**Figure 6:**
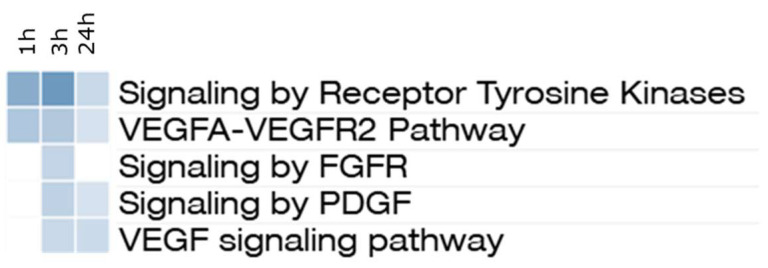
Down-regulated pathways derived from phosphorylated proteins. The down-regulated proteins enrich pathways associated with VEGF, PDGF, and FGF, mainly 3 h after the administration. The blue shades indicate the q-value.

The data from phosphoprotemic analysis (9213 phospho-peptides identified and quantified with a defined position of the PTM) showed a downregulation of specific pathways, including multiple receptor tyrosine kinase signaling implicated in the mechanism of action of Nintedanib (Figure 6). The down-regulated proteins were enriched in pathways associated with VEGF, PDGF, and FGF. Many known kinases involved in Nintedanib activity [15] emerged as players of growth factor-related pathways, such as non-receptor tyrosine kinases (Src and Lyn), MAPK kinases, and Akt. Differentially expressed phospho-peptides were subjected to activation loop analysis to determine the kinases affected by Nintedanib treatment. Phosphorylation of the activation loop is known to stabilize its conformation, thereby facilitating the transition of kinases from an inactive to a fully active state [17]. The analysis demonstrated that Nintedanib induced a reduction in phosphorylation of Mapk1 (T188) and Mapk3 (T203, Y205, T208), consistent with their functional inactivation.

### 2.4. Assessment of Nintedanib Lung and Plasma Exposure

**Figure 7:**
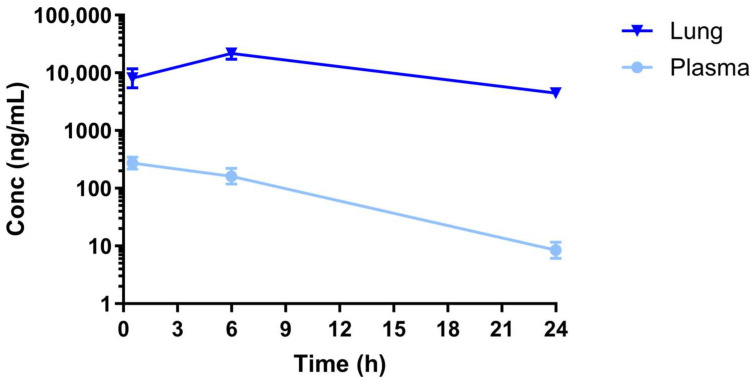
Assessment of Nintedanib exposure. Plasma and lung samples from the 28-day BLM study performed in rats orally dosed with Nintedanib at the dose of 100 mg/kg under a once daily regimen were assayed for drug exposure over the last day of treatment.

On day 28, plasma and lung samples were assayed for Nintedanib exposure (three collected timepoints: 0.5 h, 6 h, and 24 h after the last treatment; N = 3 for each time point). Data are reported in Figure 7. Plasma level of the drug at the last time point was ≈9 ng/mL (≈4800 nM). Lung levels were higher than corresponding plasma levels, with a lung/plasma concentration ratio increasing over time from 30 to ≈500. This profile is indicative of high tissue distribution and consistent with the high volume of distribution reported by the literature on Nintedanib [18,19].

## 3. Discussion

Nintedanib is an orally available, small-molecule multi-kinase inhibitor, approved for the treatment of IPF. The main therapeutic molecular targets for Nintedanib are VEGF, PDGF, and FGF kinase receptors [12,16]. Nintedanib was also approved for systemic sclerosis-associated interstitial lung disease [20] and demonstrated anti-fibrotic potential in diverse preclinical models when administered by oral gavage or by intraperitoneal and intrapulmonary route [21,22,23]. To the best of our knowledge, no dedicated analysis has been conducted in preclinical models of lung fibrosis to demonstrate in vivo Nintedanib target engagement and to evaluate its plasma and lung concentrations supporting its molecular mechanism of action. These objectives represent the primary aims of the present study.

In this study, a double administration of BLM was chosen to better mimic human IPF compared to the traditional single-dose model. This approach, supported by our previous research, induces a more severe and persistent fibrotic response, with sustained upregulation of translationally relevant ECM-related genes [24]. Additionally, as demonstrated in murine models [25], the double-hit Bleomycin rat model confirmed the presence of a cell population defined as KRT8+ alveolar differentiation intermediate (ADI) cells, which may contribute to the establishment of an aberrant tissue repair process and ECM deposition in the Bleomycin rodent models [26].

We firstly confirmed the anti-fibrotic effect of Nintedanib in a double-hit BLM rat model of lung fibrosis through the downregulation of several molecular makers (including HYP, pro-collagen 1, WISP-1, and MMP-7, Collagen type 1 and Fibronectin), and the reduction in the Ashcroft scores and of the automated fibrosis quantification determined by a machine learning algorithm (Figure 2 and Section 4). In parallel, three subsets of soluble ligands (VEGF, PDGF, and FGF2), linked to Nintedanib’s mode of action [16], were assessed in lung homogenates and plasma. Nintedanib did not modify PDGF lung levels but significantly reduced lung levels of FGF2 when compared to BLM-treated animals (Figure 4A,B). In contrast, Nintedanib significantly increased lung and plasma VEGF ligand levels compared to both BLM-treated (Figure 4C,D) and untreated (Figure 4E,F) animals, strongly suggesting the ability of Nintedanib to directly/molecularly interact with VEGF.

Proteomic analysis of lung homogenates of Nintedanib naive rats revealed a distinct effect of the drug on growth factor receptors. Although VEGF, FGF, and PDGF ligands were undetectable, the respective receptors were quantified. Indeed, PDGFR appeared not modulated; FLT1 (VEGFR1) showed a trend of increase over time, while FGFRs were downregulated, reaching statistical significance at 24 h post-drug administration, at least for FGFR3. Phosphoproteomic analysis further supported the modulation of pathways targeted by Nintedanib, highlighting the drug’s impact on downstream signaling events. Pathways analysis of phosphoproteomics data confirmed the inhibition of signaling induced by receptor tyrosine kinases, with a peak at 3 h post-drug administration. As expected, the Nintedanib-modulated growth factor signaling involved all the MAPK cascades that are believed to be major regulators of the Mapk1/Mapk3 cascade [27]. The mechanism of action of Nintedanib is further supported by the activation loop analysis, which demonstrates the downstream inactivation of kinases (Mapk1/Mapk3) following receptor engagement by Nintedanib. Phosphoproteomic data indicated that the optimal time point to observe downregulation downstream of PDGFR, VEGFR, and FGFR was 3 h post-treatment. In contrast, in the bleomycin model, animals were sacrificed 24 h after the last drug administration. This temporal difference explains why the proteomic data from the bleomycin experiment did not reveal a pronounced effect of Nintedanib on growth factor receptors’ downstream signaling. Instead, the residual molecular signature primarily reflected modulation of the VEGF ligand. These results suggest that although the levels of VEGF ligand are elevated in the lungs of animals treated with Nintedanib, the VEGF signaling pathway remains functionally downregulated, consistent with the expected inhibition of VEGF receptors by Nintedanib.

VEGF ligands and receptors are known as key regulators of normal and pathological vasculogenesis and angiogenesis [28,29]. Aberrant angiogenesis is also a prominent histopathological feature of pulmonary fibrosis [30,31]. VEGF exerts several other functions, such as induction of epithelial proliferation and prevention of epithelial cell apoptosis, both in vitro and in vivo [32,33,34]. Nevertheless, the role of the VEGF signaling pathway in the pathophysiology of IPF remains controversial, and the association of this growth factor with IPF development is poorly understood in both clinical and preclinical settings [31,34,35,36,37,38,39,40,41,42]. Stockmann et al. suggested that the discrepancy observed between various studies in the effect of VEGF inhibition on pulmonary fibrosis could be linked to a matched, protective, and detrimental effect of VEGF [43]. Furthermore, in the above studies, VEGF levels were assessed in diverse compartments (blood, Bronchoalveolar lavage (BALF), lung) from IPF and control individuals [39]. For instance, VEGF plasma levels were significantly related to radiologic fibrosis scores in patients with idiopathic interstitial pneumonias [40], whereas VEGF levels were reduced in broncho-alveolar lavage fluid from patients with IPF [44]. Accordingly, the diagnostic and prognostic roles of VEGF in fibrotic Interstitial Lung Diseases (ILDs) remain to be established [45,46,47,48]. Taken together, these findings indicate that alterations in the temporospatial expression and/or action of VEGF might impact IPF phenotypes and highlight the need for improved modeling systems to study the role of VEGF in lung fibrosis. In addition, scRNA sequencing studies and proteomics (https://www.proteinatlas.org/) have shown that VEGF ligands and receptors are also expressed in several non-endothelial IPF lung cells, including type II alveolar epithelial cells, fibroblasts, dendritic cells, macrophages, and aberrant basaloid cells [49,50].

Nevertheless, the increased VEGF lung and plasma levels, as suggested by our pharmacokinetic data, may not be able to exert their biological effects at least as long as the VEGF receptors (FLT1, KDR, and FLT4) remain hindered by “super-threshold” levels of Nintedanib.

While the mechanism behind increased plasma VEGF after VEGFR inhibitor treatment remains unclear, several clinical studies provide insight. For example, sunitinib, a multitargeted TKI inhibiting VEGFRs and other kinases, has been shown to markedly elevate plasma VEGF in metastatic renal cell carcinoma patients [51]. After the first treatment cycle, VEGF rose over threefold in 44% of cases, returning to near baseline during off-treatment periods, indicating a drug-dependent effect. Similar findings were reported by Liu et al. [52], where VEGF increased significantly during sunitinib exposure across different dosing schedules and normalized upon withdrawal. Drevs et al. [53] observed a dose-dependent VEGF-A rise with compound PTK787/ZK222584, another VEGFR inhibitor. Collectively, clinical use of VEGFR inhibitors is associated with increased circulating VEGF levels, supporting the concept of a class effect for agents targeting VEGFRs. Our current findings align closely with these clinical observations, suggesting that a comparable feedback mechanism may also operate in rodents. Main reasons suggested by the literature for this on-target effect could be explained mainly throughout two hypothesis: (1) the compensatory upregulation of VEGF, as a feedback mechanism to maintain VEGF system deeply regulated, as proposed from the above mentioned studies or (2) loss of VEGF clearance by an increased VEGFR-VEGF complex internalization, as already disclosed elsewhere [54,55]. A potential mechanism underlying the observed increased VEGF levels may involve the activation or removal of inhibitory regulators on VEGF promoter activity. To explore this, we revisited our previous research on Nintedanib’s transcriptomic profile in a rat model of bleomycin-induced lung fibrosis [56]. While that study primarily highlighted molecular effects of nintedanib in the context of lung fibrosis amelioration, RNA sequencing data revealed the upregulation of genes such as Kruppel-like factor 4 (*KLF4*) and ETS transcription factor 6 (*ETV6*), both of which are mechanistically linked to VEGF regulation and expression [57,58]. *KLF4* and *ETV6* resulted statistically up-regulated in the BLM + Nintedanib group respect BLM + Vehicle, with log2FC values of 0.771 and 1.299, respectively. Interestingly, both genes were downregulated by BLM treatment. Although preliminary, these findings might suggest a promising research direction focused on mechanisms that modulate compensatory activities within VEGF signaling after VEGF receptor blockade.

To complete the analysis, we also measured plasma and lung levels of Nintedanib, confirming a high tissue distribution, consistent with findings reported in humans [18,19]. Furthermore, plasma levels at the last time point (≈9 ng/mL, ≈4.8 µM) were comparable to the median trough concentration achieved in humans with the twice daily dose of 150 mg used in IPF patients (≈10 ng/mL). Concentrations of 10–13 ng/mL were estimated as the trough concentration producing 80% of the Emax (EC_80_) on FVC decline in the clinical setting [24]. In the present work, Nintedanib plasma and lung levels ≥ 9 ng/mL (≥4800 nM) confirm that the circulating concentrations are suprathreshold to inhibit the diverse RTKs, as reported by the 5000 nM competitive assay (Table 1). Overall, the data provided might support a valid translational significance of our approach as the results closely mimic the data obtained in IPF patients. We can reasonably assume that the exposure to Nintedanib throughout the whole BLM experiment was adequate in terms of target engagement, as confirmed by the observed anti-fibrotic effects and the modulation of FGF2 and VEGF levels by the drug. Indeed, the observed plasma concentrations were always higher than the EC_80_ observed in humans.

In summary, we demonstrated that in vivo administration of an active oral dose of Nintedanib (100 mg/kg) to BLM-treated rats results in plasma concentrations of the drug comparable to those detected in a clinical setting [18,19]. At the observed plasma concentrations, Nintedanib exerted its anti-fibrotic effect and significantly inhibited lung and plasma markers linked to its mode of action (e.g., FGF2). Conversely, we have identified VEGF as a marker significantly enhanced by Nintedanib in plasma and lung of both BLM-treated and BLM-free animals. This latter finding, as proof of target engagement, may suggest the activation of a “compensatory” positive feedback in response to the VEGF receptor blockade. To better understand the sequence of events leading to the observed increase in VEGF levels in rats following Nintedanib treatment, future research should focus on the identification of the target cells responsible for the new synthesis and release of VEGF ligands induced by Nintedanib and the main organ/system involved. scRNASeq and potential sc-proteomic data could represent the most direct starting point to address this new research line. Overall, clinical evidence from Sunitinib and PTK787/ZK 222584, together with our data on elevated VEGF levels in rats, suggests that increased circulating levels of VEGF might derive from a compensatory feedback mechanism and may serve as a dynamic biomarker indicative of effective target engagement of VEGFR modulators. In clinical settings, this parameter could help establish optimal dosing and correlate with therapeutic outcomes, as well. In non-clinical models, monitoring VEGF and related proteins in plasma not only may support efficacy evaluation and dose selection but also deepens mechanistic understanding by revealing biological pathways influenced by tyrosine kinase inhibitors. Therefore, plasma VEGF measurement represents a versatile tool bridging mechanistic research and translational applications. Whether our findings have any direct translational significance in humans remains to be elucidated. Assessing whether patients treated with Nintedanib display increased plasma levels of VEGF, compared to baseline, may reveal VEGF as an indicator of Nintedanib target engagement in clinical practice.

## 4. Materials and Methods

### 4.1. Kinase Cell-Free Assay

Kinase primary screen binding interactions of Nintedanib were assessed at Eurofins Scientific (https://www.eurofins.com). Nintedanib was screened at three ascending concentrations (100, 1000, and 5000 nM) against a selected panel of kinases based on previously reported Nintedanib mechanism of action [16]: FGFR1, FGFR2, FGFR3, FGFR4, PDGFRA, PDGFRB, VEGFR1 (FLT1), VEGFR2 (KDR), and VEGFR2 (FLT4).

### 4.2. Animals and BLM-Induced Lung Fibrosis Model

Male Sprague–Dawley (SD) rats, weighing 250–300 g (Charles River, Calco, LC, Italy), were provided with food and water ad libitum. After an acclimatization period, animals were lightly anesthetized with sevoflurane, and afterwards, were intratracheally injected with a double administration of Bleomycin sulfate (1 U/kg, BLM, Baxter Oncology GmbH, Halle (Westfalen), Germany) or equal volume of 0.9% saline solution (Veh) using a Penn Century Microsprayer (Penn-Century Inc., Philadelphia, PA, USA) at day 0 and at day 4 (Figure 1A) [59]. According to a therapeutic approach, Nintedanib (100 mg/kg/day in methylcellulose 0.5%) was orally administered for three consecutive weeks starting from day 7. To maintain all groups homogeneous, all animals received a gavage procedure with Nintedanib or its vehicle. All data are obtained by merging four independent experiments with the same protocol (see Statistical Analysis paragraph). All the groups were anesthetized by intraperitoneal injection of 200 mg/kg thiopental (pentothal sodium, MSD Animal Health Srl, Segrate, MI, Italy) and sacrificed at day 28 post-BLM. Whole lungs were removed and weighed. Left lung lobes of all animals were destined for histological analysis, while right lung lobes were utilized for assessment of fibrotic biomarkers and for quantification of Nintedanib levels. Plasma samples were obtained as well by blood collection from cardiac puncture and following centrifugation at 2000 rpm, 4 °C, for 10 min. The body condition score and Grimace Scale were used to monitor the overall health condition and to predict the actual establishment of the fibrotic lesions in the rat lungs. All the procedures involving experimental animals were reviewed and approved by the local ethics committees and authorized by the Italian Ministry of Health (authorization number: 1066/2015; approval date: 5 October 2015, and 246/2021-PR; approval date: 7 April 2021). Moreover, all procedures were performed within a certified animal facility: AAALAC (Association for Assessment and Accreditation of Laboratory Animal Care, https://www.aaalac.org/). All experiments were performed in full compliance with the European ethics standards in conformity with directive 2010/63/EU, Italian D. Lgs 26/2014, the revised “Guide for the Care and Use of Laboratory Animals” [60], and the ARRIVE guidelines (Animal Research: Reporting of In Vivo Experiments). Moreover, a group of naive animals, not receiving BLM, were treated with a single administration of Nintedanib and sacrificed as described above at different time points (0, 1, 3, and 24 h) for lung sampling and plasma collection to measure VEGF levels and for proteomic analysis.

### 4.3. Histopathology Assessment

Left lung lobes were removed and inflated with gentle infusion of 10% neutral-buffered formalin, fixed for 24 h, and then paraffin-embedded (FFPE). Longitudinal 5 μm-thick sections were collected from each sample. To assess fibrotic lesions, a score evaluation was made on Masson’s trichrome-stained slides, using a method based on the Ashcroft scale (grade 0 to 8), as described previously [61,62]. A one-by-one mm grid was applied to each section, and an Ashcroft score value was assigned to each of the squares identified by the grid, as previously described [24]. The distribution of fibrosis was characterized by grading the Aschroft score in three classes of increasing values, ranging from 0 to 2 (physiological), 3–4 (mild), 5–6 (moderate), and 7–8 (severe) [63]. Results are expressed as the mean value of the assigned score and as the frequency of distribution of 3 different classes arbitrarily identified. Stained slides with Masson’s trichrome were also scanned and acquired into the Visiopharm Integrator System (VIS; version 2017.2.4.3387) for a quantification of pulmonary fibrosis by automated analysis with the support of a VIS Analysis Protocol Package (APP) [24].

### 4.4. Markers Analysis in Lung Homogenate and Plasma

#### Enzyme-Linked Immunosorbent Assay (ELISA)

Tissues were processed as previously described [20]. Briefly, frozen right lobes were weighed and homogenized in 10 mL of ice-cold 1X PBS (10010023, Thermo Fisher Scientific, Waltham, MA, USA) per g of tissue, with gentleMACS™ Dissociator (Miltenyi Biotec, Bergisch Gladbach, Germany) and Polytron PT2500E (Kinematica, Malters, Switzerland) with the protease and phosphatase inhibitor cocktail (PPC2020, Sigma-Aldrich, Saint Louis, MO, USA). The total amount of HYP was determined with a Colorimetric Assay Kit (Sigma MAK008, USA) according to the manufacturer’s protocol. An aliquot of right lung homogenate was frozen/thawed three times and centrifuged (5000× *g* for 10 min) to obtain a supernatant cell lysate. As markers of fibrosis, procollagen-I, WISP-1, and MMP7 were assessed in lung homogenate supernatants. Moreover, three additional soluble ligand sets (VEGF, PDGF, and FGF2), linked to Nintedanib’s mechanism of action as indicated elsewhere [16], were quantified in lung and plasma. All markers were quantified by Enzyme-linked immunosorbent assays (ELISA) commercial kits: procollagen-I (ab210579, Abcam, Cambridge, UK), WISP-1 (Mouse/Rat WISP-1/CCN4 Quantikine ELISA Kit, MWSP10, Bio-Techne, Minneapolis, MN, USA), MMP7 (Rat MMP-7 ELISA Kit, NBP3-06896, Bio-Techne, Minneapolis, MN, USA), PDGF (Rat PDGF ELISA Kit, orb567597, Biorbyt Cambridge, UK), FGF2 (Mouse and Rat FGF basic/FGF2/bFGF ELISA Kit—Quantikine, MFB00, Bio-Techne, Minneapolis, MN, USA) and VEGF-A (Rat VEGF-A ELISA Kit, ab100787, Abcam, Cambridge, UK). Diluted plasma was also used to evaluate VEGF levels using a Magnetic Luminex assay (Rat premixed multi-analyte kit, LXSARM, Bio-Techne, Minneapolis, MN, USA). All procedures were performed in accordance with the supplier’s instructions. Results were expressed as picograms (pg) in right lung weight.

### 4.5. Lung Sample Preparation and nLC-HRMS/MS Conditions for Proteome Analysis

Sample preparation for label-free proteomics: lung homogenates from both the rat models described were quantified with the bicinchoninic acid assay (BCA). A portion of 300 μg of each sample was digested for 16 h with Trypsin (1:20 *w*/*w*) following the S-trap mini protocol. An amount of 10 μg of peptides from each sample was digested and used for global proteomics analysis. The group of naive animals treated with Nintedanib at different time points was also subjected to phosphoproteomics analysis. The peptides digested were enriched with TiO_2_ beads (5020–75,000, GL Sciences, Tokyo, Japan) for phosphopeptides, with a method adapted from the Easyphos platform [64]. The eluted phosphopeptides were desalted (89,870, Thermo Scientific) prior to LC-MS analysis.

Nano-LC-MSMSM: Peptide samples (1 μg) were analyzed using a Thermo Scientific Dionex Ultimate 3000 nano RSLC system coupled to a nanoESI source. Peptides were initially trapped on a PepMap100 μ-precolumn (300 μm i.d. × 5 mm, C18, 5 μm, 100 Å) in 0.1% TFA. Separation was performed on an EasySpray C18 column (75 μm × 500 mm, 2 μm, 100 Å) at 40 °C with a flow rate of 300 nL/min. A gradient from 5 to 50% acetonitrile with 0.1% formic acid over 240 min was applied, followed by 50–85% in 5 min, a 20 min wash at 85%, and 30 min equilibration. Mass spectrometry was conducted in data-dependent acquisition (DDA) mode using a top-speed approach. The most abundant ions were fragmented by collision-induced dissociation (CID), and reporter ions were detected in the Orbitrap at 30,000 resolution. The instrument operated in positive polarity mode, excluding singly charged precursors from fragmentation.

Database searching: database searches were performed with Proteome Discoverer v2.4 software (Thermo Scientific) using Sequest HT search engine and Rattus norvegicus database (2020) and contaminants. Search parameters included trypsin, permitting two missed cleavage sites, carbamidomethyl in cysteine as static modification, methionine oxidation, acetylation in protein N-terminus, and phosphorylation S/T/Y as dynamic modifications. Peptides with a q-value lower than 0.1 and an FDR < 1% were considered as positive identifications with a high confidence level.

Differentially Expressed Proteins and Pathways Analysis: The statistical analysis performed by Proteome Discoverer was used to identify differentially expressed proteins and differentially expressed phosphopeptides. All those proteins that showed a log2fold-change of at least +/−0.58 and satisfied adj *p* < 0.05 were considered differentially expressed. Significantly upregulated and downregulated proteins and phosphoproteins were employed for the functional enrichment analysis and generation of a physiological pathways map using the web-based portal Metascape 3.5. Analysis of activation loop phosphorylated sites was performed with an online tool specific for kinases

### 4.6. Assessment of Nintedanib Plasma and Lung Levels

Bioanalysis and quantification of Nintedanib in rat specimens were performed using a fit-for-purpose LC/MS/MS method developed internally. Plasma and lung homogenate samples (lung tissue was homogenized with a water/acetonitrile 50/50 mixture) were extracted by means of protein precipitation with 3 volumes of acetonitrile (Sigma-Aldrich Co, St. Louise, MO, USA); the supernatant was directly injected into the LC/MS/MS system.

The plasma and tissue concentrations of Nintedanib were determined using a calibration curve prepared in the corresponding blank matrix, and the accuracy of the quantitation was verified using quality controls prepared in the same matrix and back-calculated on the curve. The accuracy of the back-calculated analyte concentration was within ±15% of the calibration standard nominal value (except 20% for the lower limit of quantitation). Values falling outside these limits were discarded, but at least 75% or a minimum of 6 calibration standards were used for the curve. At least 67% of the quality control samples showed accuracy within ±15% of the nominal value, and there was at least one valid quality control sample for each concentration level. Blank plasma was prepared with Dichlorvos as stabilizer (10 µL of a 0.1 M solution in acetonitrile for each mL of plasma).

An Agilent liquid chromatography system (1260 pump equipped with 1200 autosampler) was used (Agilent, Palo Alto, CA, USA). The auto-sampler and column oven were kept at 10 °C and 30 °C, respectively. Chromatographic separation was achieved on a Phenomenex EVO C18, 50 × 2.1 mm, 2.6 μM (Phenomenex, Torrance, CA, USA) using 0.1% formic acid in water (Carlo Erba reagents S.A.S., Val De Reuil Cedex, France; Solvent A) and 0.1% formic acid in acetonitrile (Carlo Erba reagents S.A.S.; solvent B) as mobile phases. The gradient program was 0.0–0.5 min, 5% B; 0.5–3.5 min, gradient to 90% B; 3.5–5.0 min, 90% B; 5.0–6.0 min, gradient to 5% B; 6.0–10 min, 5% B.

The flow rate and injection volume were set at 0.35 mL/min and 5 μL, respectively. The HPLC system was coupled to an AB SCIEX 4000 Q-TRAP triple quadrupole mass spectrometer (AB Sciex, Foster City, CA, USA) equipped with an ESI source. The mass spectrometer operated in positive ion mode. Ion spray voltage was set at 5000 V while the temperature was set at 450 °C. Curtain Gas, Source Gas 1, and Source Gas 2 were set at 20, 30, and 35, respectively. Quantification was operated in multiple reaction monitoring (MRM) mode using the *m*/*z* transition 540.3 → 113.1 (Collision Energy: 39; Declustering Potential: 116).

### 4.7. Statistical Analysis

Data are representative of four independent experiments, with each experimental group comprising between 5 and 10 animals.

All values are expressed in mean ± SEM. One-way ANOVA test followed by Dunnett’s post hoc test was used for multiple comparisons. A *p*-value < 0.05 was considered statistically significant. Histograms and statistical analysis were performed with Graphpad Prism v. 10.1.0 (GraphPad Software, San Diego, CA, USA).

## Figures and Tables

**Table 1:** In vitro interactions between Nintedanib and selected human kinase receptors.

Target Kinase	Nintedanib Concentrations (nM)		
	100	1000	5000
FGFR1	49%	3.9%	0.1%
FGFR2	58%	16%	3.8%
FGFR3	64%	9.5%	1.3%
FGFR4	98%	55%	17%
PDGFRA	0.2%	0.1%	0.0%
PDGFRB	0.0%	0.0%	0.0%
VEGFR1 (FLT1)	1.6%	0.0%	0.3%
VEGFR2 (KDR)	0.1%	0.0%	0.0%
VEGFR2 (FLT4)	0.2%	0.0%	0.0%

Results for primary screen binding interactions are reported as “% of the control response”, where lower numbers indicate stronger interaction with the target kinase.

## Data Availability

The original contributions presented in this study are included in the article. Further inquiries can be directed to the corresponding author.
